# A Case of Linezolid Toxicity Presenting as a Sepsis Mimic

**DOI:** 10.1155/2019/2157674

**Published:** 2019-12-17

**Authors:** Rashmi Mishra, Harish Patel, Bindu Goel, Trupti Vakde

**Affiliations:** ^1^Department of Medicine, Penn Highlands Healthcare, PA, USA; ^2^Division of the Gastroenterology, BronxCare Health System, NY, USA; ^3^Department of Medicine, BronxCare Health System, NY, USA; ^4^Mercy Medical Center, IA, USA; ^5^Division of the Pulmonary and Critical care Medicine, BronxCare Health System, NY, USA

## Abstract

Linezolid is an efficacious and well tolerated antimicrobial but can have serious adverse effects including myelo-suppression, serotonin syndrome, neuropathy, hypoglycemia, liver dysfunction, and lactic acidosis. The side effects are generally duration dependent; linezolid use is not recommended for more than 28 days. *Case.* A 59-year-old female presented with malaise, loss of appetite, and altered mentation. She had multiple medical comorbidities and required long-term anticoagulation with warfarin for venous thromboembolism. She had multiple medication allergies. Prior to admission, she was on linezolid for cellulitis of foot due to Methicillin-resistant *Staphylococcus aureus* (MRSA). On physical exam, she was drowsy and required endotracheal intubation for airway protection. Initial laboratory parameters showed lactic acidosis, thrombocytopenia, supra-therapeutic coagulation profile, low blood glucose, and transaminitis. Her altered mentation was due to hypoglycemia. The interaction with warfarin led to altered coagulation profile. She developed shock and vasopressors were initiated. Given her presentation, she was managed as severe sepsis. There were no active infectious foci attributing to decline of her clinical status. Linezolid was discontinued and she was managed with intravenous polymyxin B, aztreonam, and vancomycin. Her hemodynamic status improved within one day. She was extubated on Day 5 of her presentation. Her laboratory parameters showed gradual improvement over 12 days after discontinuation of linezolid. Retrospective evaluation revealed linezolid toxicity as possible cause of presentation. Linezolid toxicity can present as sepsis mimic and should be considered as a differential diagnosis while managing sepsis with other antimicrobial agents.

## 1. Introduction

Linezolid is an oxazolidinedione antibiotic approved for use against serious Gram-positive infections, including methicillin-resistant *Staphylococcus aureus* (MRSA) and vancomycin-resistant enterococcus faecium [1]. It is generally well tolerated, but can have serious adverse effects including myelosuppression [2], thrombocytopenia [3], serotonin syndrome [4], neuropathy [5], liver dysfunction [6], hypoglycemia, and lactic acidosis. However, rare but potential warfarin interaction leading to hypercoaguable state has been reported [7].

Antibiotics are used for the management of the bacterial infection. The antimicrobial induced adverse events, like lactic acidosis, can be confused with presentation of severe sepsis. It is imperative to recognize the cluster of adverse events to guide optimal treatment. A rare triad of toxicity of the hypoglycemia, lactic acidosis, and acute pancreatitis from linezolid has been reported [8]. We present a novel case of the linezolid toxicity presenting with a rare constellation of adverse events in the form of thrombocytopenia, lactic acidosis, hypoglycemia, and warfarin toxicity. It is imperative to include linezolid toxicity in differential diagnosis of sepsis and use alternative antibiotics for the management of possible bacterial infection.

## 2. Case

A 59-year-old female presented with malaise and low appetite for one day. Her family brought her to emergency room (ER) in view of altered mentation. Her medical comorbid conditions included coronary artery disease, hypertension, diabetes mellitus, and multiple venous thromboembolism requiring long-term anticoagulation with warfarin, chronic renal insufficiency, and steroid dependent asthma.

Few weeks prior to her index presentation, she was hospitalized for foot infection. Her evaluation did not reveal osteomyelitis. She developed cellulitis near the ulcer and wound culture revealed growth of Methicillin resistant *Staphylococcus aureus* (MRSA). The antibiotics susceptibility revealed sensitivities to vancomycin and moxifloxacin. Infectious disease consultation was obtained and in view of the allergy to moxifloxacin and renal insufficiency, 3 weeks of the linezolid was recommended. She was initiated on intravenous linezolid 600 mg twice daily. She was followed up in the infectious disease ambulatory clinic. She was followed up in the anticoagulation monitoring clinic, and she did not require any alteration in warfarin dose. Glucose monitoring diary maintained by visiting nurse service revealed a controlled diabetes state. There was interval improvement in cellulitis and foot ulcer. She presented to our ER few days after the clinic assessment.

She had multiple medication allergies. She was allergic to azithromycin, penicillin, moxifloxacin, aspirin, ibuprofen, and ketorolac. She had unclear history of allergy to vancomycin. However, further evaluation during index hospitalization revealed the reaction to be rate dependent infusion reaction.

Her initial vitals were blood pressure of 90/46 mm Hg, oxygen saturation of 97% on room air, heartrate of 110 beats per minute, and she was afebrile. Her physical examination was significant for respiratory distress and drowsiness. The foot exam revealed interval healing of the foot ulcer. There were no meningeal signs. Neurological exam did not reveal any focal deficits. Her abdomen was soft and nontender. Cardiac auscultation did not reveal any new murmur.

Initial laboratory parameters showed lactic acidosis, thrombocytopenia, supra-therapeutic international normalized ratio (INR), low blood glucose, transaminitis, and normal fibrinogen levels (Table 1).

Imaging studies did not reveal any infective foci. There was no intracranial bleeding on Computerized tomography (CT) of the head. X-ray chest at the time of admission revealed clear lung fields with no pulmonary infiltrates. Ultrasound of abdomen revealed acalculus gallbladder and normal dimension of the common bile duct.

Given her altered mentation she required intubation and mechanical ventilation. In view of prior MRSA infection she was managed initially for sepsis. The initial impression of her presentation was sepsis, and required further evaluation for source of infection. Infection disease consultation recommended trough monitored intravenous vancomycin along with polymyxin B and aztreonam. Interdisciplinary discussion between clinical pharmacy, infectious disease consultant, and intensivist recommended further evaluation of records for vancomycin allergy versus allergic reaction. Patient received polymyxin B and aztreonam. She further developed shock. Her periphery was cold; despite fluid resuscitation she required the vasopressor for a brief period. The clinical pharmacist recommended vancomycin use, and her hemodynamic status improved with one day. Lactic acidosis can be from hypotension at presentation, but the improvement was not in conjunction to recovery of shock. This late recovery of serum chemistry led to investigate alternate etiology for lactic acidosis. Infectious disease follow-up recommended vancomycin levels to guide subsequent dose and bacterial cultures for titration of antibiotics. However, her blood culture and wound culture from infection site were negative for any bacterial growth. Both cultures were obtained at time of presentation prior to antibiotics administration. Hematology consultation opined that the hypercoaguable state was warfarin induced rather than sepsis induced. A normal fibrinogen level supported this conclusion. She completed the course of linezolid and hence it was not given at time of admission. She was extubated on Day 5 of admission. Her laboratory parameters showed gradual improvement over 12 days after discontinuation of linezolid (Table 1). Given the absence of septic foci, the clinical scenario supported the presentation to be a constellation of adverse events from linezolid and she did not require the additional antibiotics therapy.

## 3. Discussion

Linezolid binds to bacterial RNA and prevents formation of a functional 70S initiation complex which is important for the bacterial translocation process. It has been hypothesized that due to the similarities between human and bacterial ribosomes [9], linezolid inhibits the human mitochondrial ribosome by a similar mechanism. Henceforth, the deficiency of mitochondria encoded proteins can lead to lactic acidosis [10–12]. A longer duration of therapy of more than 6 weeks, has been associated with higher rates of lactic acidosis [1]. A systemic review of 47 cases of linezolid identified male gender as a risk factor for development of lactic acidosis, rather than duration of antimicrobial use and patient age [13]. Sepsis, thiamine deficiency, and liver cirrhosis have been associated with the higher incidence of the linezolid associated lactic acidosis [14]. Tissue hypoxia leads to increase in lactate production. Hypotension affects the lactate clearance and hence leads to hyperlactate state [15]. Although, hypo perfusion can be a cause for lactic acidosis in our patient. Lactic acid levels improve with sepsis management, correction of hypotension, and it is correlated with better prognosis [16, 17]. In our case, the lactate recovery did not correlate to shock management and achievement of re-perfusion status. Lactic acid recovery with linezolid toxicity is prolonged and its can take more than 10 days for the lactate levels to normalize [18]. Metformin is also known to cause lactic acidosis, specifically in patients with the renal and hepatic insufficiency [19]. Metformin can limit mitochondrial cellular respiration leading to lactic acidosis [20]. However, our patient was not on metformin. Hence, possible explanation of a prolonged plateau to normalization of lactate level can be explained by linezolid toxicity.

The literature has depicted an association of linezolid with myelosuppression [3, 14, 21, 22]. No increased risk of agranulocytosis or other irreversible blood dyscrasias was found [21]. More than 2 weeks of therapy has been found to be a risk factor for linezolid induced thrombocytopenia [21, 22]. Thrombocytopenia secondary to linezolid is reversible upon discontinuation of drug [22]. Patients with renal impairment may develop higher plasma linezolid concentration, and hence more likely to develop thrombocytopenia as result of the linezolid toxicity [3, 14]. Vitamin B6 supplementation has been reported to reverse linezolid associated cytopenia [21]. Frequent blood cell count monitoring is suggested for individuals on more than two weeks of the linezolid therapy.

Linezolid related nonselective monoamine oxidase (MAO) inhibition could induce hypoglycemia [23]. This phenomenon is further supported by the higher incidence of hypoglycemia in the individuals on MAO and insulin or sulfonylurea concurrently [24, 25].Our patient was on insulin for the management of her diabetes, but there was no evidence for the use of MAO inhibitors. The bacterial infection can lead to brittle diabetes status, but we did not identify any active bacterial infection while managing her acute presentation. Her diabetes was well controlled at the time of ambulatory visit assessment prior to ER presentation. Medications with dopamine (D2) receptor agonist activity, like bromocriptine, are utilized for antihyperglycemic effect and can cause side effects of hypoglycemia. Linezolid may increase the levels of dopamine via its MAO inhibition activity, hence leading to hypoglycemia [23]. Another mechanism of the hypoglycemia is from linezolid induced mitochondrial dysfunction leading to pancreatic beta-cell related insulin secretion dysregulation [8].

Initial reports of the potential interaction between warfarin and linezolid surfaced in 2015 [7]. Linezolid can have the antimicrobial activity against Bifidobacterium, hence compromising the vitamin K production in the gut [7]. It is hypothesized that linezolid can decrease vitamin K production and potentiate the warfarin toxicity. Our patient had a stable coagulation profile on a stable dose of warfarin until linezolid initiation and there were no other interaction attractable to the hypercoagulable state.

Transaminitis related to linezolid (drug) induced liver injury has been well reported [6, 10]. The liver histopathology in these cases reveals diffuse microvesicular hepatosteatosis [6]. Ischemic hepatitis can be alternative explanation, but would require the clinical acumen to differentiate between the two etiologies. In ischemic hepatitis there is delayed peaking of bilirubin with ALT/LDH ration of lower than 1.5 [26]. In index case, the bilirubin levels peaked at presentation and ALT/LDH ratio was 1.8. Sepsis can be an alternate explanation, but a clear infectious etiology could not be demonstrated.

The hemodynamic status in our patient improved rapidly. However, the resolution of other parameters like transaminitis, thrombocytopenia, and hyperlactatemia required multiple days leading to prolonged hospitalization.

In view of pancytopenia and neuro-toxicity the European Medicines Agency (EMEA) and U.S. Food and Drug Administration have suggested to limit linezolid use to 28 days [27, 28]. Linezolid remains an effective antimicrobial for MRSA related infections. A prolonged duration (>4 weeks) of linezolid use, though not well tolerated, has been reported for management of bone infection [29]. In our patient, in view of her multiple drug allergies and renal insufficiency linezolid remains the preferred antibiotic. We faced challenges in management of possible MRSA infection in view of unclear history of vancomycin allergy. Mitochondrial genetics has been studied in linezolid toxicity , specifically for those related to shorter duration of therapy [30]. In presented cases, mitochondrial genetic can explain presentation of lactic acidosis. However, etiology of other side effect was unrelated.

## 4. Conclusion

Prolonged use of the linezolid has been associated with lactic acidosis, hypoglycemia, thrombocytopenia, and interaction with warfarin. This case brings to light the need for physicians to be aware of linezolid toxicity being a sepsis mimic during course of management. Alternative antimicrobial agents should be considered for management of suspected bacterial infection.

## Figures and Tables

**Table 1:** Demonstrates laboratory parameters at the time of admission and the improvement after discontinuation of linezolid.

Parameters	Day 1	Day 3	Day 5	Day 12	Day 114
Hemoglobin (g/dL)	8.4	6.6	7.9	7.9	
WBC (k/*µ*L)	16	2.9	6.7	21.1	
Platelets (k/*µ*L)	26	17	11	190	
INR	8.4	1.9	1.6	1.4	
Creatinine (mg/dL)	3.3	3.1	2.3	1.0	
AST ( U/l)	1289	559	101	346	19
ALT (U/l)	897	688	418	669	33
LDH (units/ml)	479				
Bilirubin (gm/ml)	2.1	1.3	1.4	0.9	
Lactic acid (mMol/L)	12.4	6.5	4.4		
Blood gas (pH)	7.06	7.4	7.38		
Blood glucose (mg/dL)	54	139	207	260	
Fibrinogen (mg/dL)	225				

## Abbreviations

MRSA: Methicillin-resistant *Staphylococcus aureus*
INR: International normalized ratio.
